# The Precision of All-on-Four Implant Position Recorded from Three Different CBCT Machines

**DOI:** 10.1055/s-0044-1788613

**Published:** 2024-07-23

**Authors:** Osamah Alsulimani, Abdulrahman Alhaddad, Mosa Altassan, Asmaa Bukhari, Lulu Munshi, Ghalia Sabir

**Affiliations:** 1 Department of Oral Diagnostic Sciences, Faculty of Dentistry, King Abdulaziz University, Jeddah, Saudi Arabia; 2 Department of Oral and Maxillofacial Prosthodontics, Faculty of Dentistry, King Abdulaziz University, Jeddah, Saudi Arabia; 3 Internship program, Faculty of Dentistry King Abdulaziz University, Jeddah, Saudi Arabia

**Keywords:** CBCT, All-on-Four, precision, degree of deviation

## Abstract

**Objective** To investigate the dimensional discrepancy and degree of deviation of All-on-Four implant position between different cone-beam computed tomography (CBCT) machines.

**Materials and Methods** Four implants (4.5 × 10 mm Superline II, Dentium, South Korea) were placed in an All-on-Four style in an artificial mandible. The jaw was radiated 30 times using three different CBCT machines (Rainbow CT, Dentium; Veraview X800, Morita, Japan; Planmeca Viso G3, Planmeca OY, Finland). A total of 30 Digital Imaging and Communications in Medicine (DICOM) files were exported, *n* = 10. All-on-Four implants from each DICOM file were segmented and exported as an STL file (three-dimensional image) using Blue Sky Plan software (version 4.12.13/Blue Sky Bio, United States). All-on-Four implant zone dimensions ( *X* , *Y* , and *Z* axes) and the total degree of deviation between All-on-Four implants per CBCT machine were measured using Autodesk Meshmixer software (version 3.5.474/California, United States). The data distribution's normality and variances' equality were tested with Shapiro–Wilk's and Levene's tests, respectively ( *p* -value < 0.05). Data were analyzed using Brown–Forsythe one-way analysis of variance and Tamhane's post hoc tests to compare the differences between the groups ( *p* -value <0.05).

**Results** The respective *X* , *Y* , and *Z* mean dimensions of the All-on-Four implant zone were: Dentium (34.95, 14.71, and 9.97); Morita (34.88, 14.74, and 10.56); and Planmeca (34.73, 15.15, and 12.33). Significant differences between CBCT machines were found in all axes ( *p* -value < 0.05); however, the *Z* -axis had the most differences. Notably, Planmeca exhibited the highest standard deviation (SD) in all axes (0.16–0.35), exhibiting the lowest consistency in the CBCT machines' readings. The Dentium exhibited the lowest deviation in the implant position, with the lowest SD (0.61). A significant difference in the total degree of deviation was spotted when only Morita was included in the comparison ( *p* -value < 0.05).

**Conclusion** This study's findings are of significant importance as they reveal that the implant position recorded from the CBCT machines was most discrepant in the buccolingual dimension ( *Z* -axis). Planmeca exhibited the least implant-dimensional accuracy of the CBCT machines, while Dentium exhibited the highest implant position accuracy. These results could significantly impact the choice of CBCT machine for implant placement, especially since an accurate CBCT image is crucial for digital implant planning.

## Introduction

The cone-beam computed tomography (CBCT) machine is a well-established adjunctive diagnostic, virtual simulation, and treatment planning tool with numerous clinical applications in multiple disciplines. This device can produce three-dimensional images with a significant reduction in radiation exposure, a shorter scan time, and lower costs compared with conventional CT. 1 The information obtained from CBCT scans allows the measurement of bone density, height, and the buccolingual width of the alveolar bone at any specified jaw location 2 ; allows a comprehensive understanding of accurate jaw dimensions and anatomical structures, decreasing potential risks and dramatically enhancing predictability of treatment results 3 ; offers multiple advantages compared with traditional two-dimensional radiography, including a lack of superimposition, 1:1 measurement, absence of geometric distortions, and three-dimensional (3D) display. 4 The efficiency of CBCT machines is on the rise, and there is also a rapid growth in current software packages designed for processing, managing, and analyzing 3D images. Moreover, CBCT is quickly spreading and has become the imaging modality for planning implant placement. 5 Virtual treatment planning includes using software (primary or third party) available with CBCT images, which allows virtual implant planning that can be transferred to the surgical site directly by using image-guided navigation or indirectly via the construction of a surgical guide. 6

CBCT images are appraised to allow highly accurate and reliable linear measurements. Furthermore, the accuracy of reformatted CBCT images is affected by several factors. These include the characteristics of the machine itself (e.g., nominal resolution and image quality), radiation exposure (kV, mA, and the number of basis images, the software for reconstruction and dimensional measurements, patient motion artifacts, and the limitations of the clinician's interpretation. To determine the best application of CBCT in dentistry, it is necessary to analyze the accuracy of the data acquired related to distance measurements. 7 CBCT is considered to yield accurate volumetric data, and its multiplanar reformatted images are generally regarded as reliable for linear measurements. However, it is important to acknowledge that the accuracy of these measurements may be influenced by a variety of factors, including metallic artifacts, patient motion, and device-specific exposure parameters. 1 8 However, there is no universal standard considering the exposure geometry and parameters for CBCT scanners. 3 As previously mentioned, the presence of metallic objects will likely show artifacts and will thus jeopardize the image quality and hamper the visualization of the implant–bone surface. 9 10 Therefore, it is still questionable whether the measurement performed near dental implants is accurate. 11 Parameters of the CBCT devices may be of utmost importance to achieve better image quality and enhance evaluation accuracy. 11 12 The accuracy of measurements must be defined as CBCT imaging is commonly used to determine linear dimensions in different clinical dental applications. 1 The anatomic radiographic fidelity of bone structures and the accuracy of linear measurements are pivotal for primary preoperative implant planning and even more so when applied in guided implant surgery imaging. 13 Guided surgery systems are not perfectly precise, which can cause deviations in both horizontal and vertical directions from the planned implant site to the virtual position before the surgery. 14

A plethora of CBCT machines are available in the dental market. These different machines use different acquisition times, resulting in different effective radiation doses for the patient. They differ in three significant properties: field of view, voxel size, and focal point. The success and survival of implants crucially depend on thorough diagnosis and treatment planning, which can be achieved by using a CBCT machine. 2 The increasing need for dental implants to replace missing teeth requires a sensitive technique to obtain highly accurate alveolar and implant site measurements to assist in treatment planning and avoid damage to the adjacent vital structures during surgery. 6 The rapid advancement of CBCT scans and computed-aided implant planning programs is believed to enhance CBCT technology and improve implant placement accuracy. 15

Knowledge of the measurement accuracy of CBCT images using software programs is essential for validating their use for examining implant sites and for understanding and associating all possible sources of error in the multistep and complex implant surgery process. Such complex treatment planning sequences may result in potential errors, so it is crucial to address the issue of possible deviations between the preoperative plan and the postoperative implant location. 16

Most *in vivo* clinical studies rarely quantify measurement accuracy, as this would require an intervention to control the radiographic measurements themselves. 17 Thus, this study aims to investigate the reliability of different available CBCT machines for digital implant planning by retrospectively evaluating the precision of position and dimension of previously placed implants recorded from those CBCT machines. Significant deviation from reality will affect implant planning as distances measured from the CBCT images are crucial, such as proximity to adjacent implants, teeth, and vital structures. Two null hypotheses were formulated for this study. The first hypothesis was that there would be no significant difference in the dimensions of the All-on-Four implant zone between different CBCT machines, and the second hypothesis was that there would be no significant difference in the total degree of deviation of the All-on-Four implant position between different CBCT machines.

## Materials and Methods

### Sample Preparation and CBCT Imaging

An artificial mandibular jaw (Straumann, Switzerland) was selected to have four implants (4.5 × 10 mm Superline II, Dentium, South Korea) placed in an All-on-Four concept. The implants were placed freehand following the manufacturer's instructions. Three different CBCT machines of variable voxel size were chosen for this experiment (Rainbow CT, Dentium, South Korea; Veraview X800, Morita, Japan; Planmeca Viso G3, Planmeca OY, Finland). CBCT machines' properties are listed in Table 1 . A putty mix (Easy Putty, Variotime, Germany) was used to stabilize the jaw on the CBCT mount for imaging. The jaw was radiated 10 times per CBCT, following the manufacturer's instructions. A total of 30 Digital Imaging and Communications in Medicine (DICOM) files were exported, *n* = 10.

**Table 1:** CBCT machines' properties

Properties	Rainbow CT	Vera view ×800	Planmeca Viso G3
FOV	Ø 5 × 5, 16 × 10, 16 × 18 mm (stitching)	Ø 170 × 120 mm Ø 170 × 50 mm Ø 140 × 100 mm Ø 140 × 50 mm Ø 100 × 100 mm Ø 100 × 50 mm Ø 80 × 80 mm Ø 60 × 60 mm Ø 40 × 40 mm	Ø 40 × 50 mm
Focal spot (mm)	0.5	0.5	0.5
Voxel size	300 µm (high 200 µm)	80, 125, 160, 200, 250 µm	150–300 µm
Tube voltage	60–100 kVp	60–90 kV	90 kV

Abbreviations: CBCT, cone-beam computed tomography; CT, computed tomography; FOV, field of view.

### Implant Segmentation

All-on-Four implants from each DICOM file were segmented and exported as an STL file (3D image) using Blue Sky Plan software (version 4.12.13/Blue Sky Bio, United States). The segmentation was manually performed, and the density was set at 3,000 HU. Since the jaw was radiated every time in the same position per CBCT machine, this ensures perfect alignment on all DICOM files of each CBCT machine, hence, accurate measurement. The segmentation was then exported as an STL file for analysis.

### Dimensional Discrepancy

The analysis was performed using Autodesk Meshmixer software (version 3.5.474/California, US). Analysis was applied by measuring the All-on-Four implant zone using the *X* , *Y* , and *Z* axes in the software to investigate the discrepancy in each dimension. The *X* -axis represents the mesiodistal dimension, the *Y* -axis represents the apico-occlusal dimension, and the *Z* -axis represents the buccolingual dimension. The recorded readings included 90 readings, 10 for each CBCT machine in each dimension.

### Total Degree of Deviation Analysis

**Fig. 1:**
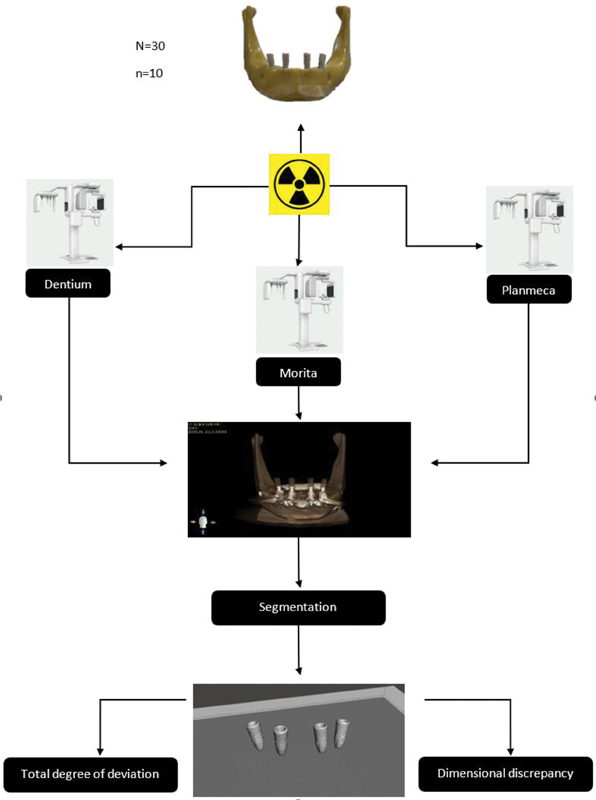
A diagram of the methodology.

The total degree of deviation of All-on-Four implants was measured to evaluate the precision of the All-on-Four implant position of each CBCT imaging. Measurement was done using Autodesk Meshmixer software (version 3.5.474). The deviation was measured between the implants' 3D images of each CBCT machine. Forty-five deviation readings were recorded for each CBCT machine, for a total of 135 readings. A diagram summarizing the methodology is shown in Fig. 1 .

### Statistical Analysis

The data distribution was tested for normality, and the variances were tested for equality with Shapiro–Wilk's and Levene's tests, respectively ( *p* -value < 0.05). The data were only normally distributed in the *Y* -axis values ( *p* -value > 0.05), and variances of the values were not equal either in the implant zone analysis or in the degree of deviation analysis ( *p* -value < 0.05); hence, Brown–Forsythe one-way analysis of variance and Tamhane's post hoc tests were chosen to compare the differences between the groups ( *p* -value <0.05). The analysis of statistics was performed by utilizing IBM SPSS 22 (SPSS Inc., Chicago, Illinois, United States).

## Results

**Fig. 2:**
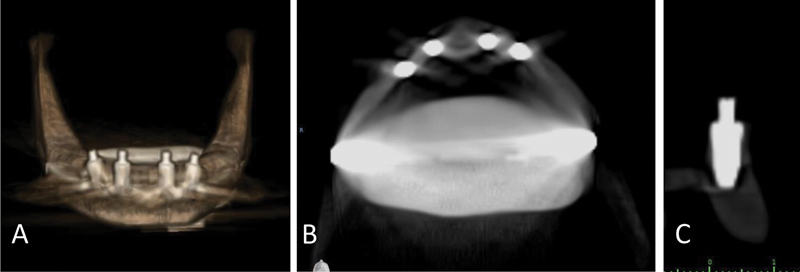
Cone-beam computed tomography images of the specimen taken by Planmeca. ( **A** ) Three-dimensional image; ( **B** ) horizontal section; and ( **C** ) implant cross-section.

Despite maintaining a constant jaw position and machine setting for 10 exposures, there was a noticeable variation in the amount of distortion present in the Planmeca CBCT and Dentium images. Morita exhibited the highest degree of distortion, followed by Dentium and Planmeca, resulting in a longer segmentation process. Morita and Dentium images boasted high resolution and clear implant surface details, resulting in simplified segmentation. Conversely, Planmeca images exhibited significantly lower resolution as illustrated in Figs. 2 3 to 4 .

**Fig. 3:**
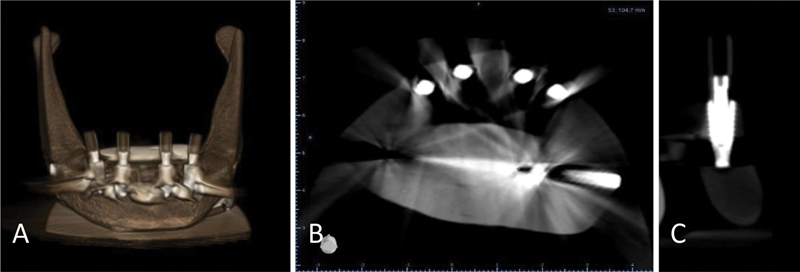
Cone-beam computed tomography images of the specimen taken by Morita. ( **A** ) Three-dimensional image; ( **B** ) horizontal section; and ( **C** ) implant cross-section.

**Fig. 4:**
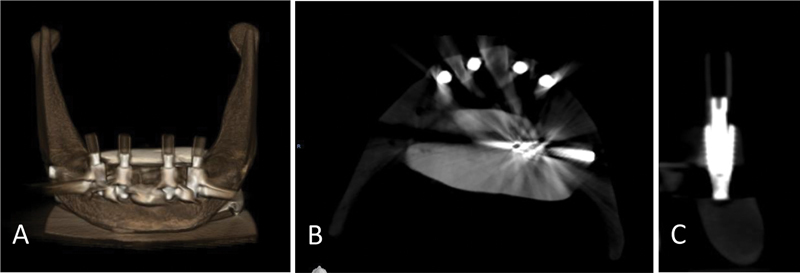
Cone-beam computed tomography images of the specimen taken by Dentium. ( **A** ) Three-dimensional image; ( **B** ) horizontal section; and ( **C** ) implant cross-section.

### Implant Zone

**Fig. 5:**
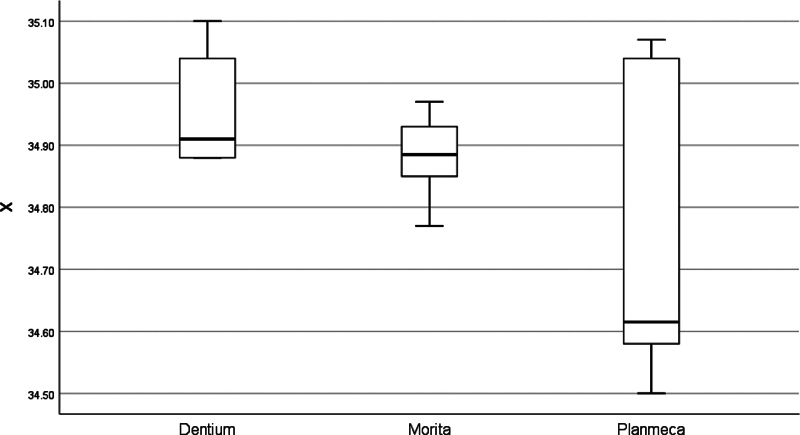
Box plot of the All-on-Four implant zone analysis in the *X* -axis of the tested cone-beam computed tomography machines.

The respective *X* , *Y* , and *Z* mean dimensions of the All-on-Four implant zone were: Dentium (34.95, 14.71, and 9.97); Morita (34.88, 14.74, and 10.56); and Planmeca (34.73, 15.15, and 12.33) ( Table 2 ). Planmeca readings exhibited the least consistency in all axes (standard deviation [SD] 0.16–0.35). Significant differences between CBCT machines were found in all axes ( *p* -value < 0.05); however, the *Z* -axis had the most discrepancy ( Table 3 ). Only the Planmeca–Dentium comparison was significant in the *X* -axis; Morita–Planmeca and Planmeca–Dentium comparisons were significant in the *Y* -axis; the *Z* -axis showed significant differences in all comparisons. Figs. 5 6 to 7 illustrate the distribution of the values and means and spot any outliers. Only two outliers were spotted, one on the *Y* -axis and one on the *Z* -axis.

**Table 2:** Descriptive statistics of All-on-Four implant zone analysis

Axis	CBCT machine	*N*	Mean	Standard deviation
*X*	Dentium	10	34.95	0.08
	Morita	10	34.88	0.06
	Planmeca	10	34.73	0.23
	Total	30	34.85	0.16
*Y*	Dentium	10	14.71	0.07
	Morita	10	14.74	0.01
	Planmeca	10	15.15	0.17
	Total	30	14.87	0.23
*Z*	Dentium	10	9.97	0.02
	Morita	10	10.56	0.09
	Planmeca	10	12.33	0.35
	Total	30	10.95	1.04

Abbreviation: CBCT, cone-beam computed tomography.

**Table 3:** Means comparison of implants zone analysis

Axis	CBCT machine		Mean difference	Standard error	Significance
*X* (mesiodistal)	Dentium	Morita	0.07	0.03	0.11
	Morita	Planmeca	0.15	0.07	0.20
	Planmeca	Dentium	0.22	0.07	0.04 a
*Y* (apico-occlusal)	Dentium	Morita	0.03	0.02	0.49
	Morita	Planmeca	0.40	0.05	0.00 a
	Planmeca	Dentium	0.44	0.06	0.00 a
*Z* (buccolingual)	Dentium	Morita	0.59	0.03	0.00 a
	Morita	Planmeca	1.77	0.11	0.00 a
	Planmeca	Dentium	2.36	0.11	0.00 a

Abbreviation: CBCT, cone-beam computed tomography.

a The mean difference is significant at the 0.05 level.

**Fig. 6:**
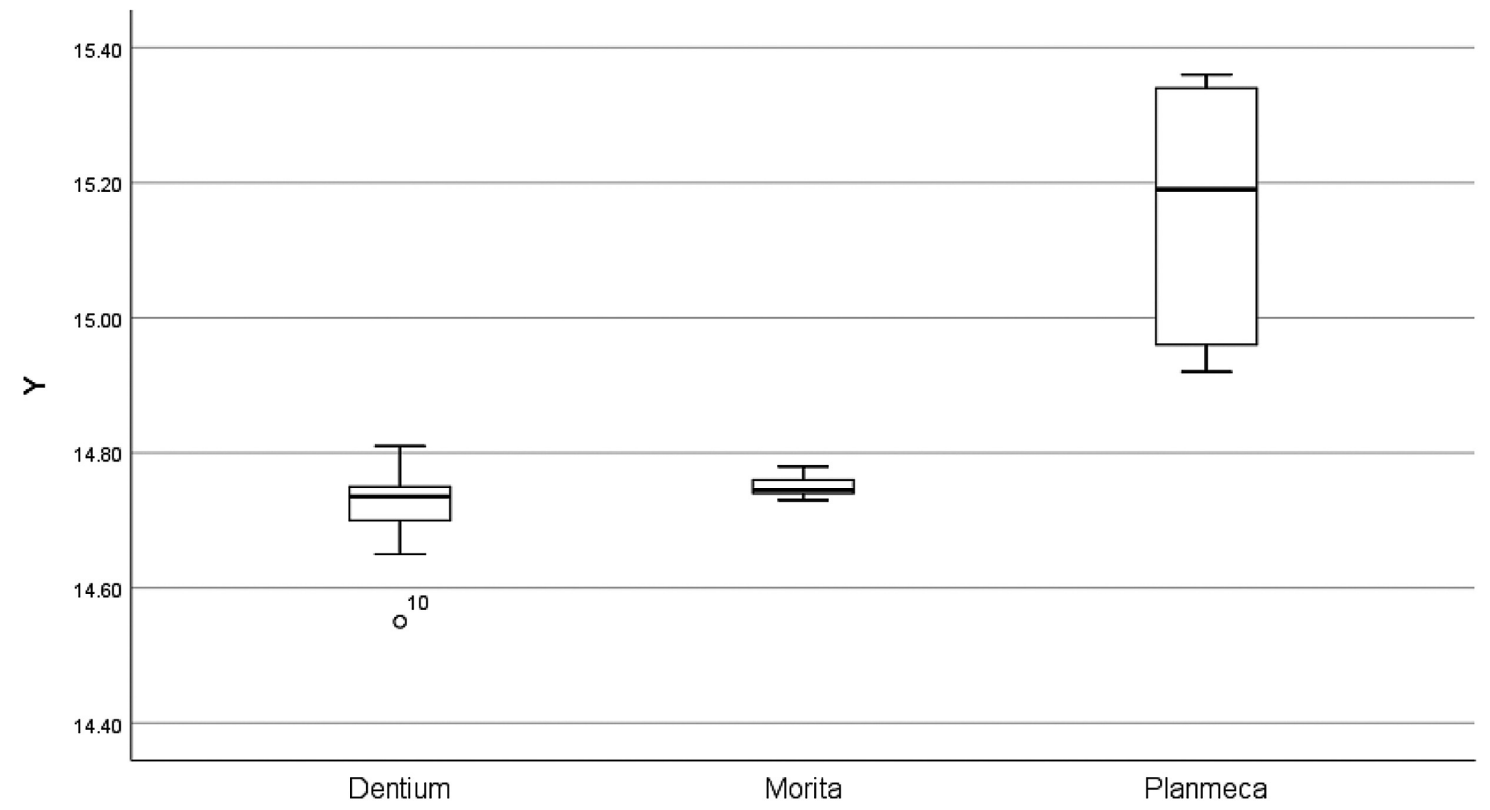
Box plot of the All-on-Four implant zone analysis in the *Y* -axis of the tested cone-beam computed tomography machines. One outlier was spotted in Dentium.

**Fig. 7:**
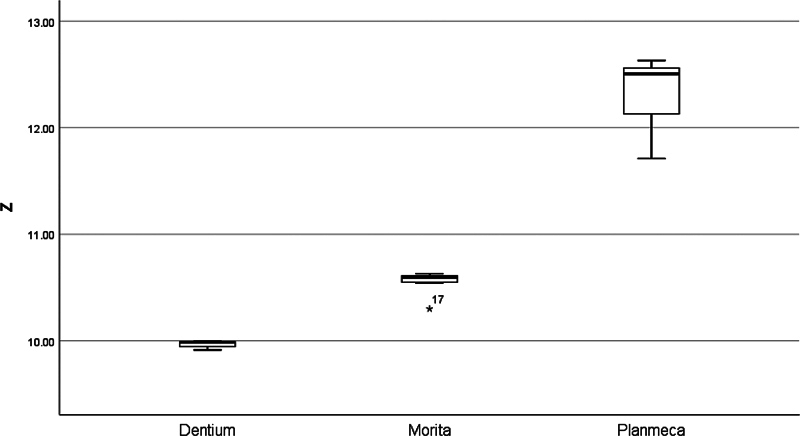
Box plot of the All-on-Four implant zone analysis in the *Z* -axis of the tested cone-beam computed tomography machines. One outlier was spotted in Morita.

### Degree of Deviation

**Fig. 8:**
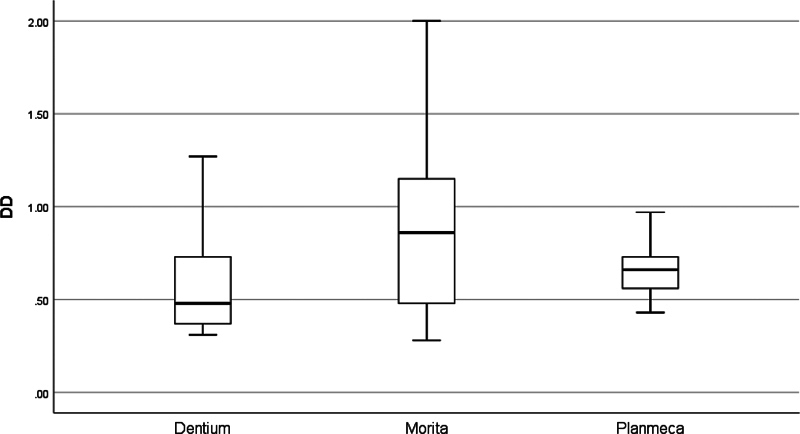
Box plot of the All-on-Four implant total degree of deviation of the tested cone-beam computed tomography machines.

As shown in Table 4 and Fig. 8 , Dentium exhibited the lowest deviation in the implant position (0.61), while Morita showed the worst (0.85); Planmeca had the most consistent readings (SD 0.13). A significant difference in the total degree of deviation was spotted when only Morita was included in the comparison ( Table 5 ).

**Table 4:** Descriptive statistics of total degree of deviation analysis

CBCT machine	*N*	Mean	Standard deviation
Dentium	45	0.61	0.32
Morita	45	0.85	0.40
Planmeca	45	0.66	0.13
Total	135	0.71	0.32

Abbreviation: CBCT, cone-beam computed tomography.

**Table 5:** Means comparison of total degree of deviation analysis

Machine	Mean difference	Standard error	Significance
Dentium–Morita	0.24	0.07	0.00 ^a^
Morita–Planmeca	0.19	0.06	0.01 ^a^
Planmeca–Dentium	0.04	0.05	0.73

a The mean difference is significant at the 0.05 level.

## Discussion

The authors have found variations in the accuracy between the tested CBCT machines. Differences were spotted in the All-on-Four implant zone dimensions and the total degree of deviation between the different CBCT machines; thus, both null hypotheses were rejected. These findings influence implant planning, which relies mainly on the accuracy of CBCT readings, especially when the digital approach is favored. Errors are still inevitable in digital implant planning; however, trivial errors should not affect the outcome significantly, especially when they are counted during the plan. Reflection 3D readings are of significant influence on the success of digital planning and guided implant placement.

There are many articles evaluating CBCT accuracy; however, based on this, the authors have decided to generate a novel method for assessing the CBCT of previously placed implants retrospectively by evaluating the discrepancy in the three dimensions of the overall implant zone and also the total degree of deviation. The analytical interpretation of All-on-Four implant zone includes the following: readings recorded from the Planmeca machine were remarkably deviated and less consistent in all dimensions ( *X* , *Y* , and *Z* axes) compared with the other CBCT machines; Morita exhibited the most consistent values through all axes; the means of Morita and Dentium were closer to each other in general in all axes compared with that of Planmeca; the *Z* -axis showed the least intramachine discrepancy (narrowest SDs), followed by the *Y* and *X* axes, respectively; however, it showed the most intermachine discrepancy (widest SDs) ( Table 2 ; Figs. 5 6 7 ). It can be noticed that when the implant zone means comparison was significant, Planmeca and Dentium were involved, except in the *Z* -axis, where all comparisons were significant ( Table 3 ). The total degree of deviation analysis showed that Dentium exhibited the lowest deviation in the All-on-Four implants position, with the most consistent readings. In contrast, the Morita machine exhibited the highest deviation, with the least consistent readings ( Table 4 , Fig. 8 ). A significant difference in the total degree of deviation was spotted when only Morita was included in the comparison ( Table 5 ). Upon comparison of the two analyses, it is evident that Morita presents the least dimensional discrepancy. However, it is worth noting that it also exhibited the least favorable readings regarding implant position. Such variations hold the potential to result in clinical complications. As such, it is recommended to exercise caution and maintain a 2-mm safety margin near adjacent anatomic structures when using CBCT.

The following studies shed light on the accuracy of linear measurements obtained from various CBCT machines and highlight the importance of maintaining safe distances while placing implants. Azhari (2019) 7 evaluated the amount of linear measurement inaccuracy of four different CBCT machines compared with clinical measurement. His study showed that three CBCT machines did not exceed a clinically acceptable threshold of 1 mm when compared with each other. Similarly, Kosalagood et al (2015) 3 investigated the accuracy of linear measurements from multiple CBCT devices and found that the radiographs slightly underestimated the actual distance when compared with physical measurements. Wanderley et al evaluated dimensional changes in dental implants using 13 CBCT devices, each set to specific scanning protocols. They found that the visualization of the implant's dimensional changes varied across the CBCT devices and scanning protocols. Specifically, they observed an increase in diameter ranging from 0.27 to 1.04 mm. 18 Finally, a recent study explored the differences among four types of 3D X-ray machines used for implant planning and found that each machine had deviations from the exact measurements, emphasizing the importance of maintaining safe distances while placing implants. 19 The investigations made could potentially hold significant weight for clinicians and researchers who rely on CBCT machines for implant planning and treatment purposes.

Several previous studies also showed that the linear measurements of CBCT images from other CBCT machines underestimate the actual distance. Underestimation is considered clinically safer than overestimation as it will preserve the vital structures when placing the dental implants. It was indicated that the difference in voxel size has no effect on the accuracy of linear measurements. 20 For example, Bohner et al 20 conducted a CBCT study of dry mandibles using voxel sizes of 0.2, 0.25, and 0.4 mm, revealing no statistical difference between the image measurements. The study suggested protocols using CBCT images with large voxel sizes (0.3 and 0.4 mm) were preferable for evaluating linear measurements for implant treatment planning due to the lower radiation dose. Therefore, the study's authors recommend that the protocol that uses the lowest effective dose is preferable for linear measurements in multiple implant planning. Additionally, in the work of Waltrick et al, 21 the accuracy of linear measurements and visibility of the mandibular canal on CBCT images were evaluated using varying voxel sizes. The experiment involved scanning 12 dry human mandibles using voxel sizes of 0.2, 0.3, and 0.4. The results indicated an average SD error between measurements on images and direct measurements of 0.23 ± 0.20 mm. Additionally, the CBCT measurements underestimated direct measurements in 390 cases. On the other hand, in 2022, the Kehrwald et al's 22 study reported no substantial variation in measurements obtained with different voxel sizes. Their study was performed to investigate the effect of voxel size on CBCT images used for dental implant planning. This was done by utilizing synthetic human mandibles with different degrees of bone resorption. Digital calipers were used for each mandible to measure the bone thickness and height. It may be recommended that larger voxel images (0.40 mm) be used when necessary for bone thickness and height measurements without negatively impacting the patient's clinical planning quality.

Accurately measuring CBCT 3D images can be challenging due to metal distortion. This study has encountered this issue. It was found that while Planmeca images had high distortion levels, CBCT images from the same machine exhibited noticeable variability in distortion levels. On the other hand, Morita images displayed clearer implant threads, but higher distortion levels resulted in a more time-consuming and difficult segmentation process. Research by Gurjar et al in 2024 23 analyzed the precision of CBCT in implant-supported prostheses and investigated metal artifacts in the presence or absence of implants or prostheses. The study assessed accuracy and artifacts at three points on the buccal and lingual cortical plates on the mandible's body near the crest and base, using physical and radiographic measurements. The results showed that CBCT artifacts were most prominent in full-arch prostheses, while single implants with a prosthesis produced the least artifacts. Although metal in implants or implant-supported prostheses can negatively impact the accuracy of peri-implant area assessment, the study suggests using lower voxel integration scales in the presence of implants or implant prostheses for more precise measurements. These findings are significant as they will aid in developing better strategies to minimize metal distortion and enhance the precision of CBCT 3D image measurements.

It is important to recognize that the segmentation process is subject to limitations due to the variability in CBCT machines, potentially leading to variable degrees of metal distortion and introducing potential bias. Also, as there was no reference file, the authors could not test how accurate the implants' positions were compared with reality. The alignment of segmented implants with digital analogs extracted from a digital impression of the implants could have provided additional reliability to this study. As a result of metal distortion, the distortion of implant surface details was unavoidable, compromising the accuracy of segmentation and potentially impacting precise alignment. It is worth considering that these limitations may compromise the accuracy of the recorded measurements.

Overall, these studies have contributed to our understanding of the accuracy and precision of linear measurements obtained through CBCT imaging. They highlighted the importance of selecting and using the appropriate CBCT device to obtain reliable and accurate measurements. Such knowledge can be helpful in enhancing the quality of diagnosis, treatment planning, and patient care.

## Conclusion

After analyzing the data, it can be concluded that Planmeca had the least dimensional accuracy among the tested CBCT machines. The most significant difference between the machines was in the buccolingual dimension ( *Z* -axis), while the smallest difference was in the mesiodistal dimension ( *X* -axis). In terms of All-on-Four implant position accuracy, Dentium had the highest accuracy, while Morita had the lowest. However, there was no statistically significant difference between the accuracy of Dentium and Planmeca machines. This new information can aid clinicians in achieving a more predictable outcome when precise full arch implant placement is crucial.
